# P-362. Outbreak of neonatal sepsis in a maternity hospital in Kyrgyzstan, 2022-2023

**DOI:** 10.1093/ofid/ofae631.563

**Published:** 2025-01-29

**Authors:** Perizat Baibolotova, Dilyara Nabirova, Lilya Bekturova, Dinara Otorbaeva, Nasyat Kemelbek kyzy, Venera Alymkulova, Roberta Horth

**Affiliations:** Jeti-Oguz Interdistrict Center for Disease Prevention and State Sanitary and epidemiological supervision, Karakol, Ysyk-Kol, Kyrgyzstan; CDC Central Asia office, Almaty, Almaty, Kazakhstan; Jeti-Oguz Interdistrict Center for Disease Prevention and State Sanitary and epidemiological supervision, Karakol, Ysyk-Kol, Kyrgyzstan; Department of Disease Prevention and State Sanitary and Epidemiological Supervision, Bishkek, Kyrgyzstan, Bishkek, Bishkek, Kyrgyzstan; Central Asia Field Epidemiology Training Program, Bishkek, Kyrgyzstan, Bishkek, Bishkek, Kyrgyzstan; Central Asia Field Epidemiology Training Program, Bishkek, Kyrgyzstan, Bishkek, Bishkek, Kyrgyzstan; US Centers for Disease Control and Prevention, Dulles, Virginia

## Abstract

**Background:**

Sepsis is a leading cause of neonatal mortality and morbidity worldwide, notably in Kyrgyzstan. In January 2023, six neonatal sepsis deaths were reported within two weeks at a 157-bed maternity hospital, which usually has < 2 cases/month. We investigated to identify sources of infection and risk factors.

**Table 1.**
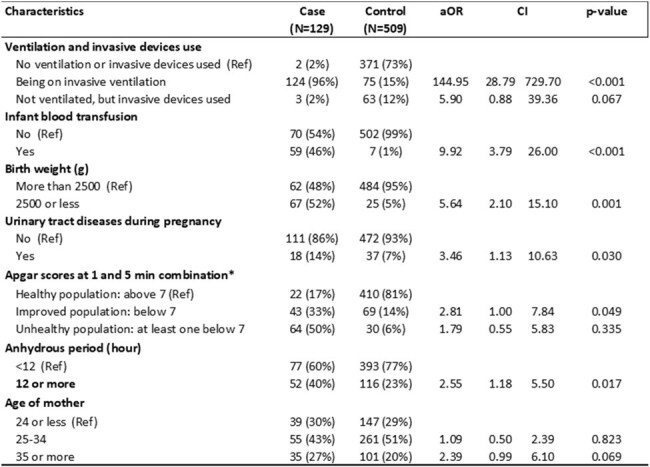
Factors associated with neonatal sepsis in a maternity hospital in Kyrgyzstan, 2022-2023 *AOR- adjusted odds ration; ** CI- confidence interval; ***1. Healthy population (Reference group): Apgar scores at 1 and 5 min above 7; Unhealthy population: Apgar scores at 1 min below 7 and at 5 min below 7; Improved population: least one below 7

**Methods:**

A concurrent case-control study included infants (< 28 days old) born at the hospital in January 2022-February 2023 and diagnosed with sepsis. Controls met same criteria and were born +/- 1 day from cases without sepsis. In February 2023, we collected rectal, nasopharyngeal, and high-touch environmental areas swabs in a single-day cross-sectional sample of hospitalized case-patients and mothers for culturing and MALDI-TOF. We used logistic regression to identify factors associated with sepsis.

**Figure 1.**
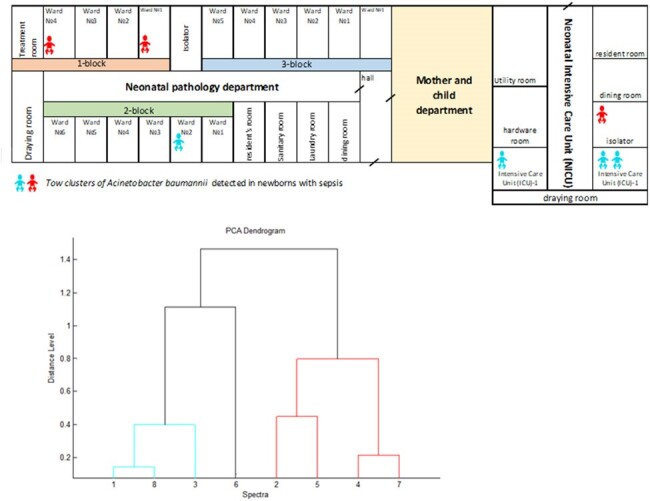
Two clusters of Acinetobacter baumannii detected among newborns with neonatal sepsis in a maternity hospital in Kyrgyzstan, 2022-2023

**Results:**

The study comprised 129 cases and 509 controls. More cases than controls weighed < 2500g at birth (52% vs 5%, p< 0.01), had anhydrous interval of ≥12 hours (43% vs 29%, p< 0.01), had Apgar scores of < 7 at 1 minute (83% vs 20%, p< 0.01), were more likely to receive both ventilation and an invasive device (98% vs 1%, p< 0.01) and to be admitted to the intensive-care unit (ICU) (82% vs 17%, p< 0.01) (Table 1). Sepsis was associated with birth weight of ≤2500g vs greater (adjusted odds ratio: 5.6; 95% confidence interval: [2.1-15.1]), infant blood transfusion vs no (9.9 [3.8-26.0]), use of ventilation and invasive device vs no (144 [28.8-729.7]), Apgar scores at 1 and 5 minutes < 7 vs greater (2.8 [1.0-7.8], urinary tract disease in mother vs not (3.5 [1.1-10.6]), and anhydrous interval ≥12 hours vs less (2.6 [1.2-5.5]). *Acinetobacter baumannii* resistant to cephalosporins, aminoglycosides, and fluoroquinolones was detected in 8/8 pharyngeal swabs from case-patients in two ICU rooms, forming two distinct clusters (Figure 1). *Acinetobacter baumannii* was not detected in environmental sampling, but the hospital had conducted deep cleaning shortly before the environmental team arrived.

**Conclusion:**

Resistant *Acinetobacter baumanni* likely contributed to the outbreak. The hospital conducted environmental cleaning, replaced non-disposable instruments (cannulas, masks, tubes) and updated infection control practices. Neonatal sepsis cases dropped sharply to < 2/month by May 2023.

**Disclosures:**

**All Authors**: No reported disclosures

